# Regional variations in nephrology trainee confidence with clinical skills may relate to the availability of local training opportunities in the UK: results from a national survey

**DOI:** 10.1007/s10157-022-02228-7

**Published:** 2022-05-07

**Authors:** Haresh Selvaskandan, Jyoti Baharani, Rizwan Hamer

**Affiliations:** 1 John Walls Renal Unit, University Hospitals Leicester NHS Trust, Leicester, UK; 2 Department of Cardiovascular Sciences, Mayer IgA Nephropathy Laboratories, University of Leicester, Hodgkin Building, Lancaster Road, Leicester, LE1 7HB UK; 3 Department of Renal Medicine, Birmingham Heartlands Hospital, University Hospitals of Birmingham, Birmingham, UK; 4 Department of Renal Transplantation and Nephrology, University Hospitals of Coventry and Warwickshire, Coventry, UK

**Keywords:** Survey, Nephrology training, Professional development

## Abstract

**Background:**

The United Kingdom offers a standardised training program for nephrology fellows. However, local training opportunities vary resulting in mismatches between trainee interests and accessible opportunities. This may impact trainee confidence, satisfaction, and future service provision.

**Methods:**

A survey assessing confidence with key procedures and sub-specialities was disseminated. Associations with region of training were probed using Chi square tests, with significance set at *p* < 0.0008 following a Bonferroni correction. Results were compared to trainee views on available opportunities for development.

**Results:**

139 responses were received (32% response rate, demographics representative of the UK nephrology trainee cohort). Procedural independence varied from 98% for temporary femoral vascular catheters to 5% for peritoneal dialysis catheters (PDIs). Independence with inserting tunnelled vascular catheters varied with region (*p* < 0.0001). Trainees expressed a desire for formal training in kidney ultrasound scanning and PDIs, corresponding with procedures they had least opportunity to become independent with. Trainees felt least confident managing kidney disease in pregnancy. Suggestions for improving training included protected time for garnering sub-speciality knowledge, developing procedural skills and for experiencing practice in other nephrology units.

**Conclusions:**

A mismatch between trainee interests and professional development opportunities exists, which may threaten trainee autonomy and impact patient care particularly with regards to peritoneal dialysis. Provisions to facilitate trainee directed development need to be made while balancing the rigors of service provision. Such measures could prove critical to promoting trainee well-being and preventing attrition within the nephrology workforce.

## Introduction

The United Kingdom (UK) operates a healthcare system that is largely nationally standardised and equitable [1]. Accordingly, nephrology services are delivered in congruence with national and international guidelines [2, 3], with audits encouraged to standardise healthcare provision where possible. Despite these measures, it is inevitable that healthcare delivery may vary regionally, influenced by factors including proximity to specialist centres and the skillsets of local health care professionals (HCPs) [4–6].

While local variations in HCP skillsets are necessary for a well-rounded service, regional variations could create inequitable training environments. Locally available opportunities may not always match trainee (fellow) interests, leading to systematic variations in skillsets determined not by individual choice, but by region of training. Where such mismatches exist, autonomy in professional development is lost. This could reduce morale, increase burnout, and at worst, lead to poorer patient care [7–10]. Such disparities are critical to address, especially in the context of the on-going COVID-19 pandemic, which has negatively impacted trainee morale and increased the risk of attrition within the UK nephrology workforce [11].

To gain insight into regional variations in trainee experience, we delivered a survey to all UK nephrology trainees. We confirmed a mismatch between trainee interests and readily available opportunities for development, discovered regional variations in confidence with key procedural skills, and found patterns with regards to sub-speciality knowledge that require addressing at both national and local levels.

## Materials and methods

An online survey was developed by the authors (supplementary material). The survey was anonymous and unincentivized. This approach was selected to foster open and honest responses, given the aim of this study was to assess trainee confidence and perceptions of accessible training opportunities.

Distribution was approved by the University Hospitals of Coventry and Warwickshire NHS Trust’s research and development department (reference: SE026). The survey was cascaded regionally by regional representatives of the Renal SpR (Fellows) Club and training programme directors (leads for regional nephrology training in accordance with national guidance). It was also advertised on trainee electronic portfolio systems [12], by the UK Kidney Association, and through social networks.

Responses were accepted between the 1^st^ of May and 1^st^ of November 2021. Questions were grouped into; demographics, academic qualifications and interests, confidence with practical skills, confidence with nephrology sub-specialities, career plans, and suggestions for training improvements. The survey included predominantly check box questions, with optional white space questions.

Ethnicity was classified according to the UK census [13]. Regions were designated by catchments for national medical training programmes, grouped into East of England, East Midlands, London, North, Northern Ireland, Scotland, South, Wales, and West Midlands (supplementary Fig. 1). Age was collected as a discrete variable. Training grades were collected as nationally designated; specialist trainee year (ST) 3–8. Independence with procedures were assessed through binary ‘yes’ or ‘no’ questions, and opportunities to perform procedures were assessed as having performed more or less than 10 instances of a given procedure in the preceding 12 months. Confidence with sub-specialities was assessed against a 5-point scale. Response rate was calculated against all nephrology trainees registered with the General Medical Council (the UK’s professional registration body for doctors) in 2021 [14].

Quantitative data are presented using descriptive statistics with proportions. Denominators are the total number of eligible responses. Associations between procedural competency and opportunities for performing procedures with region, gender, training grade and ethnicity (pooled into White, Black/African/Caribbean/Black British, Asian/Asian British, and other) were assessed using Chi-square tests. Correlations were assessed using a Pearsons correlation co-efficient. Associations between mean confidence scores with sub-speciality topics and regions and training grades were assessed with analysis of variance (ANOVA) testing. Multiple comparisons were adjusted for using the Bonferroni correction (0.05/62), following which significance was set at *p* = 0.0008, and a trend towards significance was set at *p* = 0.008. Adjusted models were not performed due to limitations in sample size.

Free-text answers were analysed qualitatively to identify key themes as follows; (1) comments were categorised as positive/negative; (2) inductive codes were derived and applied to relevant comments using “key-words in context” and “repetition of words” techniques; (3) codes sharing similar meaning were amalgamated into subthemes and frequencies were measured [15, 16]. Themes and codes were analysed by HS.

## Results

### Demographics

139 responses were received, accounting for 32% of all UK nephrology trainees [14]. Respondent characteristics broadly matched that of the national cohort (Table 1). 52% of respondents were female compared to 51% nationally. Age groups were similarly well represented; those between the ages of thirty and forty were most prevalent (87% vs 83% nationally). The East Midlands was over-represented (15% vs 6% nationally) and London was under-represented (13% vs 22%), with the remainder relatively well matched. Those identifying as Asian/Asian British were over-represented (27% vs 5% nationally), while those identifying as Black/African/Caribbean/Black British were under-represented (5% vs 31%); however, 20% of respondents chose not to list an ethnicity. Respondents in their first year of training (ST3) were under-represented (10% vs 23% nationally). Training grades of respondents did not vary across regions (*X*^2^ [48, *n* = 138] = 42.3, *p* = 0.71) (supplementary Table 1). Representation by individual regions are described in supplementary Table 2.

**Table 1:** Demographics of survey respondents compared to all nephrology trainees registered with the General Medical Council

	Survey, *n* (%)	GMC, *n* (%)
**Total**	**139 (100)**	**429 (100)**
**Females**	**72 (52)**	**217 (51)**
**Age (mean)**	**33.8**	**–**
*50–54*	*1 (0)*	*2 (0)*
*45–49*	*0 (0)*	*6 (1)*
*40–44*	*6 (4)*	*38 (9)*
*35–39*	*39 (28)*	*143 (33)*
*30–34*	*81 (59)*	*214 (50)*
*25–29*	*10 (7)*	*26 (6)*
**Ethnicity**		
*White*	*57 (41)*	*215 (48)*
*Black*	*7 (5)*	*140 (31)*
*Asian*	*37 (27)*	*24 (5)*
*Other*	*10 (7)*	*49 (11)*
*Prefer not to say*	*28 (20)*	*18 (5)*
**Training grades**		
*ST3*	*14 (10)*	*87 (24)*
*ST4*	*36 (27)*	*86 (24)*
*ST5*	*23 (17)*	*76 (20)*
*ST6*	*34 (25)*	*65 (17)*
*ST7+*	*28 (21)*	*51 (14)*
**Region**		
*Scotland*	*16 (12)*	*44 (10)*
*North*	*24 (17)*	*99 (23)*
*East Midlands*	*21 (15)*	*25 (6)*
*West Midlands*	*12 (9)*	*37 (9)*
*East of England*	*17 (12)*	*29 (7)*
*London*	*18 (13)*	*95 (22)*
*South*	*24 (17)*	*75 (17)*
*Wales*	*3 (2)*	*15 (3)*
*Northern Ireland*	*3 (2)*	*10 (2)*
**Primary medical degree**		
*UK*	*107 (77)*	*307 (72)*
*Abroad*	*32 (23)*	*122 (28)*

The bold represents the overall cohort, and the italics represent the subgroups of the bolded cohorts

### Trainee confidence varies with procedure type and region

**Fig. 1:**
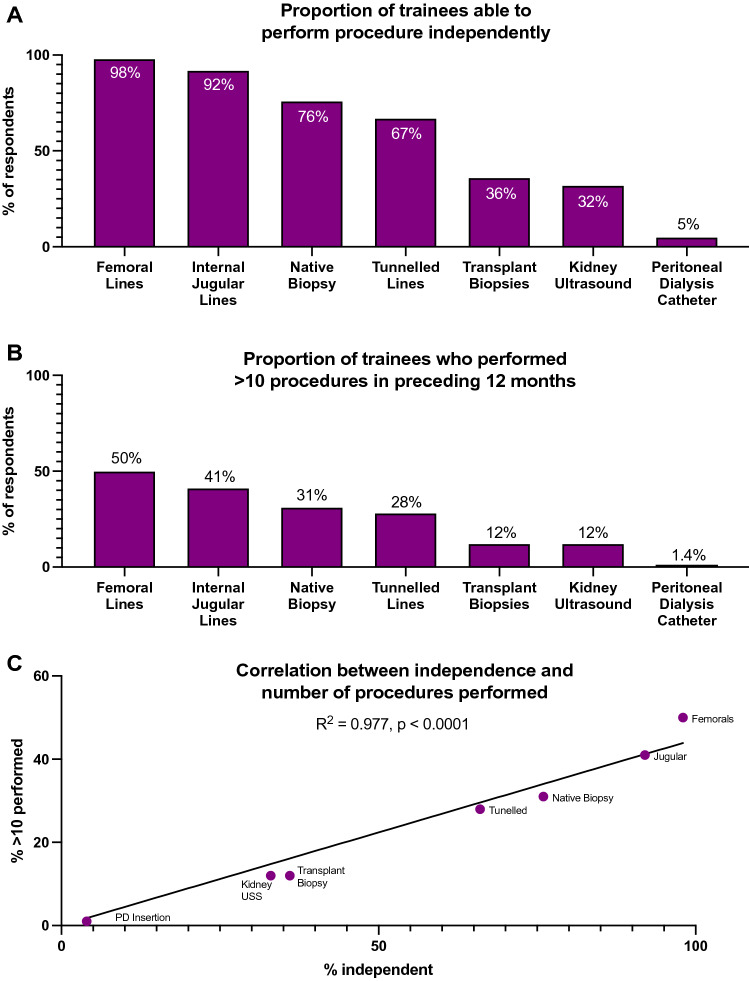
Proportion of trainees able to perform a given procedure independently (whole cohort). **A** Highlights a variation in the proportion of nephrology trainees able to independently perform a given procedure. **B** and **C** Highlights a close correlation between self-reported independence with a given procedure and the number of opportunities trainees had to perform a procedure

The proportion of trainees independent with procedures varied by intervention type (Fig. 1A). 98% felt confident inserting temporary femoral vascular catheters, 92% could insert temporary internal jugular vascular catheters and 76% could perform native kidney biopsies. The proportion able to perform other nephrology relevant procedures unsupervised were lower; 67% could insert tunnelled vascular access catheters (TVCs), 36% could perform transplant biopsies, 32% could perform kidney ultrasounds (KUS)^1^ and 5% could insert peritoneal dialysis catheters under local anaesthetic (PDIs) (Fig. 1A).

**Fig. 2:**
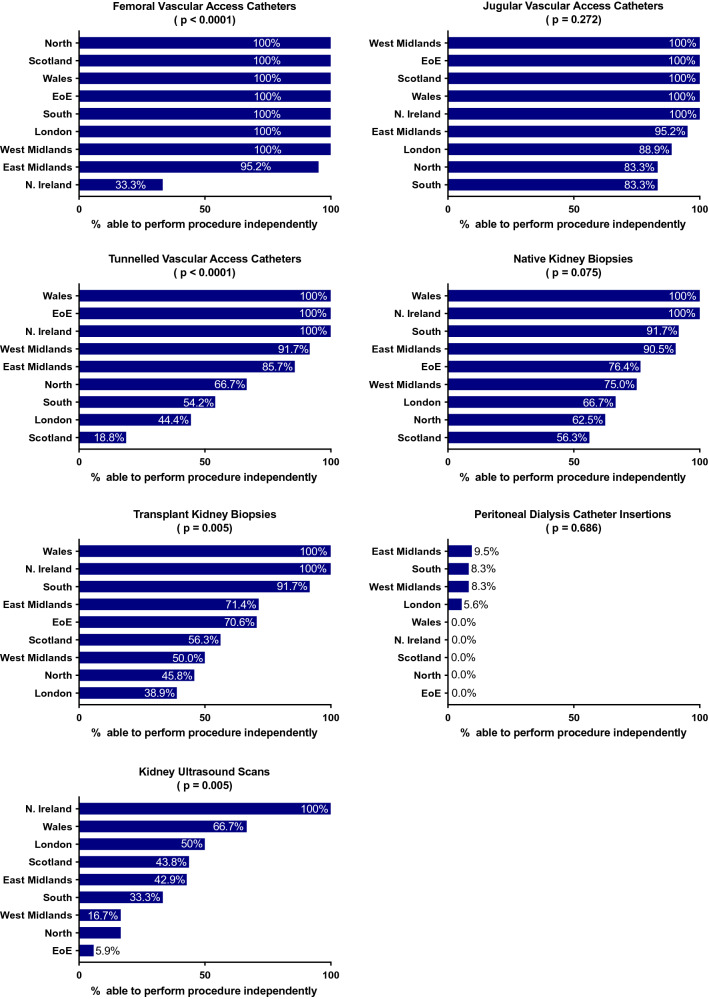
Percentage of trainees able to perform nephrology related procedures unsupervised, by region. There was a regional variation in the proportion of trainees who could insert tunnelled vascular access catheters and femoral vascular access catheters, and a trend towards regional variation with regards to transplant kidney biopsies and kidney ultrasound scans

The ability to insert femoral and TVCs varied with region (*p* < 0.0001), with a trend towards regional variation with regards to KUSs and transplant biopsies (*p* = 0.005) (Fig. 2, Table 2). No regional variations were noted for other procedures (Fig. 2, Table 2). Self-reported competency did not vary with ethnicity, gender, or training grade for any procedure, although a trend between training grade and native and transplant kidney biopsies (*p* = 0.008 and *p* = 0.002, respectively) were noted; more senior trainees felt confident performing the procedures unsupervised (Table 2, supplementary Fig. 2).

**Table 2:** Associations between self-reported procedural competence, regions, training grade, ethnicity, and gender

	Femoral vascular catheters			Internal jugular vascular catheters		
	Degrees of freedom	Chi statistic	*p* value	Degrees of freedom	Chi statistic	*p* value
Region	8	61.87	< 0.0000001*	8	9.9	0.272
Training Grade	6	3.28	0.77	6	2.68	0.848
Ethnicity	4	1.36	0.85	4	2.41	0.66
Gender	2	0.44	0.8	2	2.19	0.334

	Tunnelled vascular catheters			Native kidney biopsies		
	Degrees of freedom	Chi statistic	*p* value	Degrees of freedom	Chi statistic	*p* value
Region	8	40.5	< 0.0000001*	8	14.6	0.075
Training Grade	6	7.6	0.267	6	17.1	**0.008**
Ethnicity	4	1.6	0.812	4	3.2	0.522
Gender	2	2.72	0.256	2	2.8	0.252

	Transplant biopsies			Peritoneal dialysis catheter insertion		
	Degrees of freedom	Chi statistic	*p* value	Degrees of freedom	Chi statistic	*p* value
Region	8	21.909	**0.005**	8	5.655	0.686
Training Grade	6	20.844	**0.002**	6	7.821	0.252
Ethnicity	4	1.44	0.837	4	5.177	0.217
Gender	2	1.78	0.411	2	0.054	0.973

	Kidney ultrasound		
	Degrees of freedom	Chi statistic	*p* value
Region	8	21.9	**0.005**
Training Grade	6	11.5	0.074
Ethnicity	4	7.9	0.095
Gender	2	0.549	0.76

Bold values indicate statistical significance or a trend towards statistical significance as defined in the methods. Asterisked values indicate statistical significance

The occasions on which trainees performed procedures was also largely consistent across the UK. Temporary femoral vascular access catheters were the most accessible procedure for trainees, with 50% inserting ≥ 10 over the preceding 12 months, while PDIs were least accessible with only 1.4% inserting ≥ 10 over the preceding 12 months (Fig. 1B). This did not vary regionally, although there was a trend for regional variation with temporary jugular line vascular catheter insertions, TVCs and KUSs (*p* = 0.002, *p* = 0.006 and *p* = 0.001, supplementary Fig. 3, Table 3). There were no associations between ethnicity, gender or training grade and procedure numbers (Table 3).

**Table 3:** Associations between procedure numbers, regions, training grade, ethnicity, and gender

	Femoral vascular catheters			Internal jugular vascular catheters		
	Degrees of freedom	Chi statistic	*p* value	Degrees of freedom	Chi statistic	*p* value
Region	8	18.37	0.19	8	24.682	**0.002**
Training Grade	6	6.32	0.388	6	6.21	0.401
Ethnicity	4	1.73	0.785	4	0.49	0.974
Gender	2	7.13	0.028	2	5.87	0.053

	Tunnelled vascular catheters			Native kidney biopsies		
	Degrees of freedom	Chi statistic	*p* value	Degrees of freedom	Chi statistic	*p* value
Region	8	21.4	**0.006**	8	11.48	0.176
Training Grade	6	5.19	0.519	6	5.68	0.46
Ethnicity	4	3.05	0.549	4	2.5	0.645
Gender	2	2.96	0.228	2	7.69	0.021

	Transplant biopsies			Kidney ultrasound		
	Degrees of freedom	Chi statistic	*p* value	Degrees of freedom	Chi statistic	*p* value
Region	8	12.09	0.147	8	25.64	**0.001**
Training Grade	6	16.38	0.012	6	3.61	0.729
Ethnicity	4	6.72	0.151	4	4.11	0.392
Gender	2	6.4	0.041	2	0.226	0.893

Bold values indicate statistical significance or a trend towards statistical significance as defined in the methods

The percentage of trainees self-reporting competence with a procedure strongly correlated with opportunities to perform ≥ 10 of that procedure over the preceding 12 months (*R*^2^ = 0.977, *p* < 0.0001, Fig. 1C).

**Fig. 3:**
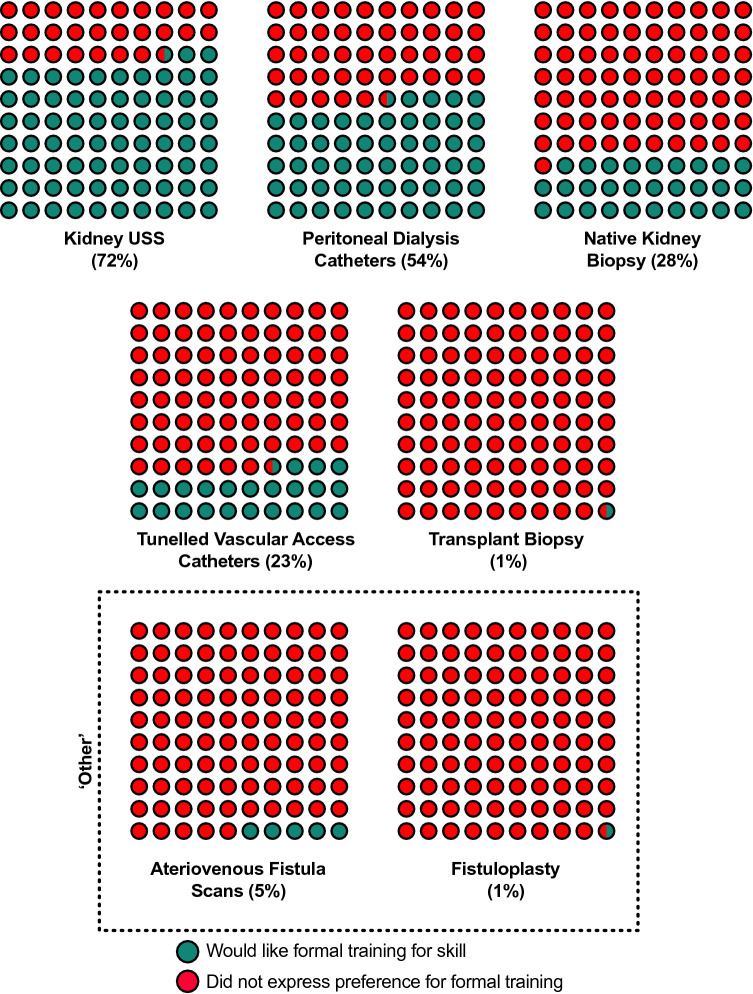
Proportion of trainees expressing preference for further training opportunities for a given procedure. The majority of trainees expressed a desire to be formally trained to perform kidney ultrasound scans and peritoneal dialysis catheter insertions

When asked what procedural skills respondents would like further training in, 72% opted for formal KUS training, and 54% selected PDIs (Fig. 3), corresponding with the two procedures trainees had the least opportunity to become independent with (Fig. 1). These preferences were broadly consistent regionally (supplementary Table 3).

### Confidence varied with sub-speciality, but not region

**Fig. 4:**
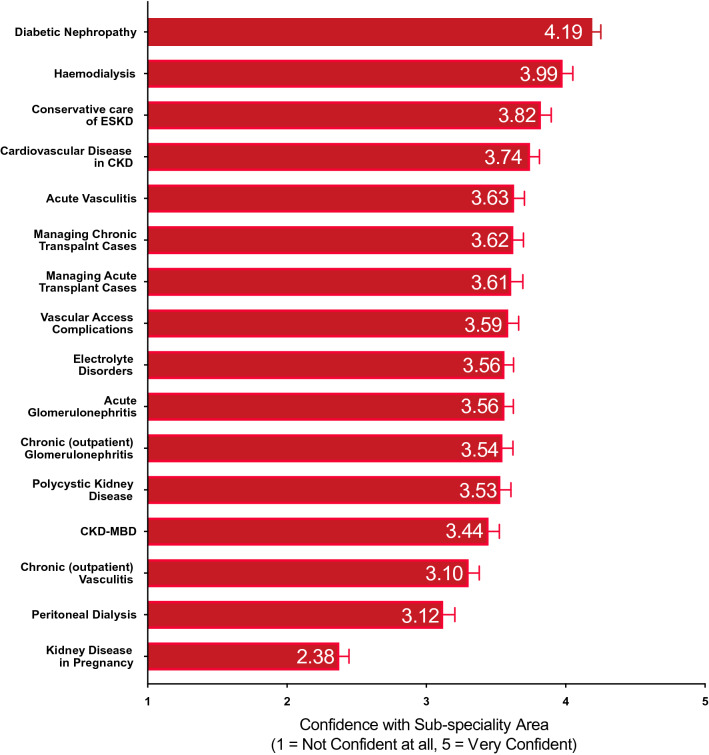
Overall Confidence with sub-speciality areas. Although there was no regional variation in the confidence trainees had with managing sub-speciality areas, there was a clear variation in confidence that was consistent across the United Kingdom. Trainees overall felt least comfortable managing kidney disease in pregnancy

Self-reported confidence with 16 nephrology sub-specialities were assessed. Trainees felt most confident managing diabetic nephropathy (mean score of 4.2, 1 = not confident at all, 5 = very confident), and least confident managing kidney disease in pregnancy (mean score 2.4) (Fig. 4). Confidence did not vary with region (supplementary Fig. 4).

**Fig. 5:**
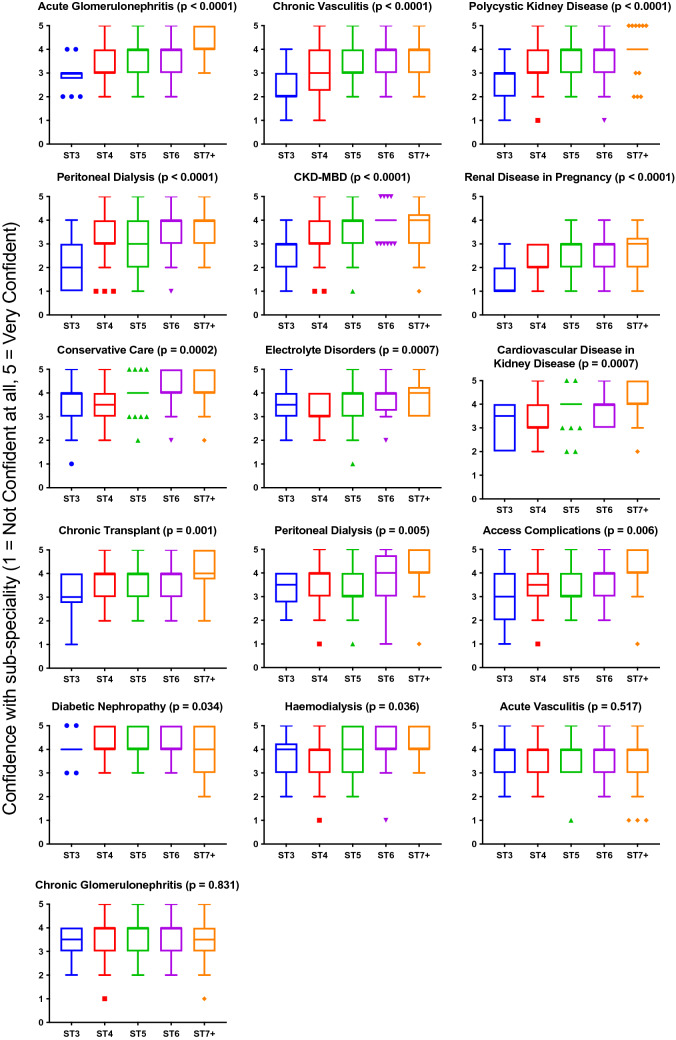
Association of training grades and confidence with sub-speciality areas. As expected, there was an association between training grade and confidence most sub-speciality areas

Confidence with sub-speciality topics varied or trended towards varying with training grade, except for chronic glomerular disorders, diabetic nephropathy, acute vasculitis, and haemodialysis (Fig. 5). No differences in confidence with topics were observed between genders or ethnicities (supplementary Figs. 5 and 6).

### Opportunities for further development

45% (43/139) of trainees reported having a higher degree (Masters level or higher), with no regional variation in this proportion (*X*^2^ [8, *n* = 139] = 4.63, *p* = 0.796). 30% of trainees were out of their training programme for further development at the time of the survey, with the most common reason cited as research (88%, 38/43), followed by leadership/management (9%, 4/43). Although there was some variation of this proportion by region (supplementary Fig. 7), this was not statistically significant (*X*^2^ [8, *n* = 139] = 9.03, *p* = 0.340). The proportion of these secondments that were funded externally (e.g., research or charitable organisations) versus the resident deanery varied by region, but did not reach significance.

### Future plans

**Fig. 6:**
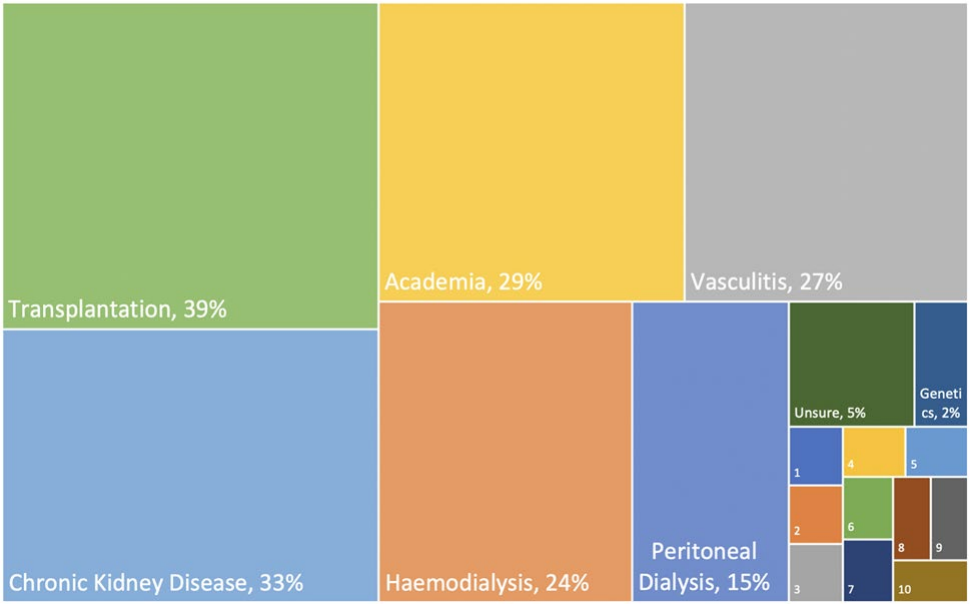
Sub-speciality of interest upon completion of training. Transplant nephrology (39%), chronic kidney disease (33%) and academia (29%) were the most popular choices expressed by trainees for sub-specialisation post training. Others included vasculitis (27%), haemodialysis (24%), peritoneal dialysis (15%), genetic kidney diseases (2%). The remaining preferences were expressed by one trainee each (1%) and are labelled 1–10 on the figure. These correspond to diabetic kidney disease, tubular diseases, care of the elderly in nephrology, acute nephrology, kidney disease in pregnancy, glomerular disorders, interventional nephrology, conservative care, onco-nephrology and cardio-renal disease. Opportunities to develop niche skill sets in these areas are lacking for trainees

The most popular areas for sub-speciality interests upon completion of training were transplant (39%), chronic kidney disease (33%), academia (29%), vasculitis (27%), haemodialysis (24%) and peritoneal dialysis (14%). 5% of trainees were unsure what their preferred area of interest would be. Genetic disease, cardio-nephrology, onco-nephrology, palliative care, interventional nephrology, glomerular diseases, electrolyte disorders, kidney disease in pregnancy, acute nephrology, care of the older person and diabetic medicine in kidney disease were opted for less than 2% of trainees each (Fig. 6). This did not vary with region (supplementary Table 4).

### Suggestions for further training experiences

The survey concluded with a free text box probing trainees’ views on how nephrology training in the UK could be improved. The theme of responses fell into the following categories; (1) protected time for research, (2) protected time to develop sub-specialist knowledge, (3) management training, (4) ultrasound training, (5) interventional procedure training and (6) time abroad or in other units. Trainees expressed a desire to have these categories built into nephrology training, citing that currently most training for sub-specialty or procedural competence was driven by self-motivation and required organising independent of their allocated service provision commitments. There were both positive and negative views towards procedural training, while some trainees were very keen to incorporate more procedural skills into formal training, others felt that this should remain optional (supplementary Table 5).

## Discussion

Although standards for nephrology training are set nationally, local delivery can vary with expertise and inclinations. Anecdotally, this creates a mismatch between trainee interests and accessible opportunities, translating to variable strengths governed by region over individual choice or service need. To explore this, we conducted a national survey; our results confirm a regional variation in trainee confidence with procedural skills, a variation in confidence with sub-speciality knowledge that is consistent across the UK, and an overarching theme of mismatches between trainee interests and accessible opportunities.

Procedural confidence is likely determined by interest, availability of training/mentoring, and opportunities for practice. Accordingly, our survey found confidence was highest with interventions performed regularly in acute settings by nephrologists (temporary vascular access catheter insertions and native kidney biopsies), but less so with those performed electively or by specialist teams (PDIs and KUS). Where trainees were able to gain independence with the latter group of procedures, free text responses revealed they were obliged to actively seek and organise training. This approach to training has inherent inequities, favouring those in regions, where working patterns permit the development of skills outside of service provision, and where available opportunities marries trainee interests. Reflecting this, regional variations in confidence with procedures including TVC insertions were noted, and nationally only 5% of respondents were able to perform PDIs unsupervised, despite over 50% expressing an interest to be formally trained with this procedure.

This mismatch between trainee interests and available opportunities can directly impact patient care. Peritoneal dialysis (PD), for instance, can be associated with improved health related quality of life [17] and can be a preferred modality for renal replacement therapy [18]. Despite this, the proportion of patients initiated on PD remains low in the UK [19]. A scarcity of training opportunities for nephrologists may partly account for this; centres, where nephrologists perform PDIs report an increased uptake of PD [20, 21]. Our survey highlights a clear mismatch between trainee interest and opportunities of training with regards to PDIs nationally, which may directly translate to improved PD uptake in the UK if addressed in the medium to long term.

Our survey also found clear variations in confidence with sub-specialities at a national level. Trainees felt least confident with kidney disease in pregnancy, followed by peritoneal dialysis. As with procedural skills, this likely reflects the relatively low case burden seen in day-to-day practice compared to other domains.

Taken together, our results highlights an opportunistic approach governing nephrology training in the UK, which trainees report primarily as ‘learning on the job’, mostly being intertwined with service provision. While this has the advantage of developing confidence managing common clinical problems, it fails to build confidence in managing rarer cases or to develop niche interests. Although those in training are expected to undergo formal appraisals every year to identify key deficiencies in skillsets, clear opportunities to pursue interests independent of service provision appear to be lacking. Reflecting this, most suggestions for training improvements fell into the category of protected time for cultivating sub-specialist knowledge or building procedural competence.

Addressing mismatches between trainee interests and learning opportunities is critical. It permits trainees to pursue their own goals, fosters individuality, and facilitates motivation and engagement with service provision and development [8, 10, 22]. This can translate to improved patient outcomes [7, 9]. At a more fundamental level, fostering individuality is fundamental to trainee well-being and job satisfaction [23, 24]. This is particularly important for preventing workforce attrition in the context of the COVID-19 pandemic, where burnout levels and plans for early retirement are at a high [11].

The trainees surveyed propose protected time for self-directed professional development; be it for developing procedural skills, sub-specialist knowledge or exploring alternative opportunities in other nephrology units. We believe these solutions to be valid. Where opportunities for developing interests do not exist locally, we support the notion of trainee ‘swaps’ to expose trainees to skillsets that not readily available in their regions. We also support the generation of clinical fellowships, where possible, to foster niche sub-speciality interests. Acknowledging funding such posts are challenging in the current environment, we propose combining these posts with clinical trial, academic, medical education or leadership and management work, for which funding sources may exist.

Our work has limitations. Although the response rate is comparable to other unincentivized, non-mandatory HCP surveys, it only accounted for 31% of UK nephrology trainees [25–28]. We opted for this approach to foster open and honest responses, uninfluenced by external pressures and reassuringly, respondent demographics broadly matched that of the UK nephrology training cohort. While those identifying as Asian/Asian British were over-represented and those identifying as Black/African/Caribbean/Black British were under-represented, we also note that 20% of our respondents chose not to declare an ethnicity. Although there were some regional variations in response rates, we could not find any systemic biases in demographic representations that would have influenced our conclusions. The responses were also all self-reported, anonymous, and confidence with sub-speciality topics were assessed with an arbitrary numerical scale, and thus may not have been interpreted in a standardised fashion. Despite this, there were indicators that our findings correlate well with current clinical practice; independence was most frequently reported with the most common clinical procedures, procedural competence correlated well with procedure numbers, there were no variations noted between those of different genders or ethnicities, and senior training grades associates with increased confidence with sub-speciality domains. Thus, given the confines of the limitations discussed, we believe the results to be broadly representative of trainee views.

Our survey highlights a clear mismatch between nephrology trainee interests and readily available opportunities. This may account for the regional variations in procedural confidence, and for national trends, such as the relatively low proportion of patients receiving peritoneal dialysis in the UK. Protected time to facilitate trainees self-development, particularly with regards to niche interests, needs to be afforded where possible while balancing the rigors of service provision. Such measures could prove critical to promoting trainee well-being and preventing attrition within the nephrology workforce.

## Footnotes

1 To perform kidney biopsies, trainees are coached to use ultrasound to identify the lower pole of the kidney and avoid surrounding structures. KUS’ involve more detailed assessments of the kidney which is beyond the scope of the competence required to perform a biopsy.
